# Childhood Rheumatic Diseases and COVID-19 Pandemic: An Intriguing Linkage and a New Horizon

**DOI:** 10.4274/balkanmedj.galenos.2020.2020.4.43

**Published:** 2020-06-01

**Authors:** Fatih Haşlak, Mehmet Yıldız, Amra Adrovic, Kenan Barut, Özgür Kasapçopur

**Affiliations:** 1 Department of Pediatric Rheumatology, İstanbul University-Cerrahpaşa Cerrahpaşa School of Medicine, İstanbul, Turkey

**Keywords:** COVID-19, hydroxychloroquine, pediatrics, rheumatology, SARS virus, tocilizumab

## Abstract

As it is known, we are all in a pandemic situation due to a novel coronavirus, officially named “Severe Acute Respiratory Syndrome Coronavirus 2” and the disease caused by the virus named “Coronavirus disease-2019”. The virus seems to has propensity to infect older male individuals with underlying disease. The clinical features were on a large scale that varies from being an asymptomatic carrier to acute respiratory distress syndrome and multiorgan dysfunction. Fever, dry cough and fatigue are the most common symptoms. Not only, the disease seems to be rare and have a milder course in pediatric age but also respiratory failure, multiorgan dysfunction, and death are extremely rare. Although several comorbidities such as hypertension, diabetes and cardiovascular diseases are defined as a risk factor for developing the acute respiratory syndrome and need for intensive care; immune-compromised situations such as rheumatic disease which require immunosuppressive treatment strikingly are not found to be a risk factor for more severe disease course. However, there is a lack of data regarding the effects of “Coronavirus disease-2019” on pediatric patients with rheumatic diseases. Additionally, there are three controversial circumstances that patients with rheumatic diseases are believed to be more likely to have viral infections like “Severe Acute Respiratory Syndrome Coronavirus 2”, on the other hand, antirheumatic drugs may have a protective and therapeutic role in Coronavirus disease-2019 and children are more unlikely to have serious disease course. Therefore, we aimed to have a contributor role for explaining this conundrum and present a bird’s eye view regarding this equivocal issue in this review.

In December 2019, a cluster of acute viral pneumoniae cases with unknown origin emerged in Wuhan, Hubei province, China (1). Most of the cases had a common exposure to the Huanan wholesale seafood market (2). Several days later, causative viral agent was analyzed with deep sequencing and indicated as a novel coronavirus, officially named 2019 novel coronavirus (2019 nCoV) (3). Coronavirus Study Group (CSG) of the International Commission on Virus Classification named this novel coronavirus “Severe Acute Respiratory syndrome Coronavirus 2 (SARS-CoV-2)” on February 11, 2020 and on the same day, the World Health Organization (WHO) named the disease caused by SARS-CoV-2, “Coronavirus disease-2019 (COVID-19)” (4). In a couple of weeks, severe COVID-19 cases were all over the world, and on the basis of disease severity and spread, finally WHO characterized the COVID-19 situation as a pandemic on March 11th, 2020 (5).

In the past two decades, addition to this current pandemic, we experienced two different coronavirus outbreaks, and previous two were named “Severe Acute Respiratory Syndrome Coronavirus” (SARS-CoV) and “Middle East Respiratory Syndrome Coronavirus” (MERS-CoV) (6). Bats are known to be reservoir hosts for SARS-CoV and MERS-CoV (7). Given the findings of genomic analyze of SARS-CoV-2, bats are assumed to be origin of also COVID-19 pandemic (8). Although intermediary animals between bats and humans are uncertain, pangolin and snakes seem to be leading suspects (2).

## PATHOGENESIS of COVID-19

It was confirmed that receptors on the human cells which spike glycoproteins (S proteins) on the SARS-CoV-2 surface binds to are angiotensin I-converting enzyme-2 (ACE 2), similar to SARS-CoV (8). It has been shown that although ACE 2 is mainly expressed in the human lungs, ACE 2 is also expressed in the gastrointestinal tract, kidney and heart (9). Human cells are being infected by the entrance of SARS-CoV-2 via S proteins binding ACE 2 followed by fusion between the virus and plasma membrane (10). After the entrance of the virus into the cells, the viral ribonucleic acid (RNA) genome is released into the cytoplasm and is translated into viral proteins followed by fusion between the vesicles containing the virus particles and plasma membrane occurs to release the virus (11).

In physiological conditions, angiotensin I acts as a substrate for 2 different enzymes; while ACE catalyzes the formation of angiotensin II, a pro-inflammatory mediator, ACE 2 catalyzes the formation of angiotensin 1-9, an anti-inflammatory mediator (12). When SARS-CoV-2 enters into cells via ACE 2 on surfaces, ACE 2 expression is downregulated and function of ACE relatively increases, resulting excessive production of angiotensin II which leads to vasoconstriction, inflammation and increases pulmonary vascular permeability (13). This mechanism seems to be one part of the possible explanation of tissue damage caused by COVID-19.

**Figure 1:**
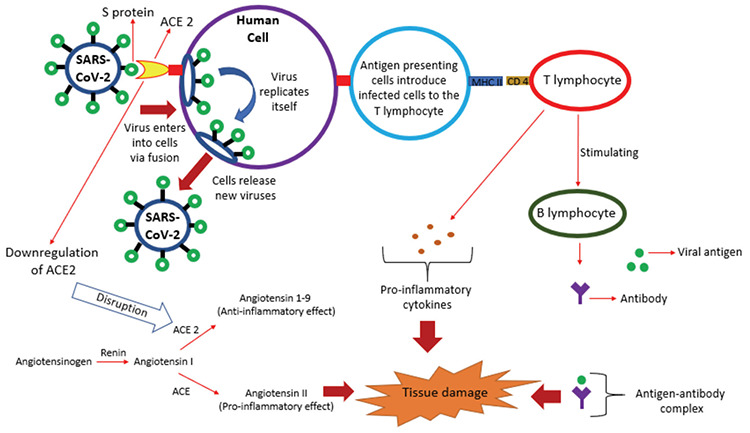
A summary of possible tissue damage mechanisms of COVID-19. SARS-CoV-2 enters into human cells via S proteins binding to ACE 2 followed by fusion between the virus and plasma membrane. Then virus replicates itself, and infected cells release new viruses. Entrance of the virus via ACE 2, downregulates the expression of ACE 2 resulting increased production of angiotensin II (a pro-inflammatory mediator) and decreased angiotensin 1-9 (an anti-inflammatory mediator). Antigen presenting cells present the infected cells to T lymphocyte, resulting excessive releasing of pro-inflammatory cytokines like IL-6 and stimulating B lymphocyte to produce immunoglobulins. Tissue damage basically occurs due to these pro-inflammatory mediators, cytokines and antigen-antibody complex.

Besides, after the entrance of SARS-CoV-2 into cells, the virus is presented to T lymphocytes, then T lymphocytes induce B lymphocytes to produce immunoglobulins, resulting antigen-antibody complexes and producing pro-inflammatory cytokines and chemokines such as tumor necrose factor (TNF) α, interleukin (IL)-1β, IL-2, IL-6, IL-10, MCP-1, MIP-1A and CCL2, resulting a significant inflammation which resemble cytokine storm syndrome (6). In order to figure out characteristics of host's immune response to the virus, immunological status of 11 SARS-CoV-2 infected patients with ARDS were analyzed by Wang et al. (14) and found that, while CD4 and CD8 T lymphocytes were significantly decreased, IL-6 was significantly increased in critically ill patients, and IL-6 should be an early predictor of severe disease. These immunological mechanisms are also another part of the possible explanation of tissue damage caused by COVID-19. Given the urgency of this pandemic situation, these mostly hypothetical explanations may be helpful for being able to plan *in vitro* medication studies. Here it is, we tried to summarize the possible explanations of tissue damages of the disease (Figure 1).

## CLINICAL FEATURES and PROGNOSIS in CHILDHOOD vs ADULTHOOD, DIFFERENCES

It was reported that, up to April 3, 2020, there were 972 303 confirmed cases and 50 321 deaths due to COVID-19 (15). Although higher rates were reported (16,17); in a study based on a large cohort of Chinese patients, overall mortality rate was announced 2.3% and tended to increase with age (18). However, we postulate that mortality rate may be overestimated, due to a significant number of asymptomatic carriers. Study of Chen et al. (16) suggests that SARS-CoV-2 has propensity to infect older male individuals with underlying disease. Regarding to need of intensive care unit (ICU) admission, although older age and to have any comorbidities were found to be risk factors in the study of Wang et al. (17), male gender was not.

The clinical features were in a large scale that varies from being an asymptomatic carrier to acute respiratory distress syndrome (ARDS) and multiorgan dysfunction (2). Fever, dry cough and fatigue were the most common symptoms; ground glass opacity is the most common screening finding and lymphopenia was found in most of the patients with COVID-19 (3,16,17).

However, the disease seems to be rare and have a milder course in pediatric age (19). Respiratory failure, multiorgan dysfunction and death were extremely rare in pediatric patients with COVID-19 (20). It has been recently reported in a huge cohort that only 1% of cases were aged under 19 years (18). In a study of Xu et al, individuals with a history of close contact with SARS-CoV-2 were tested and found that children were unlikely to be infected and display typical symptoms, compared to adults (4).

In the largest cohort of pediatric patients with COVID-19; there were 731 laboratory-confirmed and 1412 suspected cases, median age was 7 years, 94% of all cases were asymptomatic, mild or moderate and only 1 death (21). In Wuhan, 1391 children with known contact were tested, 12.3% were confirmed to have the virus, 15.8% were asymptomatic, most common symptoms were cough (48.5%), pharyngeal erythema (46.2%) and fever (41.5%) respectively, 3 had required intensive care and all three had an underlying disease (one with hydronephrosis, one with leukemia and one with intussusception) and only 1 patient died (one with intussusception) (19).

There are also confirmed newborn cases with the youngest reported a 30 hours old baby from China (22). Despite the increasing number of newborn patients with COVID-19, there is still no sufficient evidence of vertical transmission (20).

Regarding the non-severity and rarity of the disease in childhood, there are some speculations like; children have fewer outdoor activities, ACE 2 expression may be different in children, and children carry many different types of viruses in their respiratory mucosa and other viruses may limit the growth of SARS-CoV-2 (23,24). Even if children have a milder course, since they may play a crucial role in community-based viral transmission and display atypical symptoms, pediatric cases deserve a special approach.

## EVALUATION of RISK of COVID-19 in PATIENTS with RHEUMATIC DISEASE

Several comorbidities such as hypertension, diabetes and cardiovascular diseases are defined as risk factors for developing ARDS and need for intensive care (17,25). However, immune compromised situations such as a rheumatic disease which requires immunosuppressive treatment, strikingly were not found to be a risk factor for more severe disease course (26). In Italy, among 320 patients with chronic arthritis, 4 were laboratory confirmed and 4 were highly suggestive clinically for COVID-19; none of them had severe respiratory complications or died (27). In our pediatric rheumatology practice; a patient with Adenosine Deaminase (ADA) 2 deficiency and another patient with M694V homozygote mutation for Familial Mediterranean Fever (FMF), had a history of close contact with confirmed cases which they were also family members. However, both of our patients were asymptomatic and test results were negative for the virus in both. Although further information is required, to our knowledge none of the fatal cases reported were with pure rheumatic disease.

Obviously, there is a lack of data regarding effects of COVID-19 on pediatric patients with rheumatic diseases. Additionally, there are three controversial circumstances; that patients with rheumatic diseases are believed to be more likely to have viral infections like SARS-CoV-2, on the other hand antirheumatic drugs may have a protective and therapeutic role in COVID-19 and children are more unlikely to have serious disease course. Therefore, we aimed to have a contributor role for explaining this conundrum and present a bird’s eye view regarding this equivocal issue in this review.

## THE ROLE of ANTIRHEUMATIC DRUGS on the TREATMENT of COVID-19

In the treatment of COVID-19, although findings were controversial so far, antiviral agents such as ribavirin, lopinavir and ritonavir were used (28). Recently, an antiparasitic agent; ivermectin and another antiviral agent; remdesivir were found to be promising in *in vitro* studies (29,30).

Among the drugs, which are usually used in our rheumatology practice; hydroxychloroquine, an antimalarial agent mainly used in the treatment of systemic lupus erythematosus, might have a therapeutic role on the treatment of COVID-19 (31). Basis on their anti-inflammatory effects, corticosteroids and colchicine were also used (26,32). Given the pathophysiological picture of the main mechanism of lung injury in ARDS associated with COVID-19 is a cytokine storm syndrome-like, monoclonal antibodies which we often use in the treatment of patients with rheumatic diseases, are currently on focus as target-specific treatment options (14).

Although various treatment options are being used and many other are underway, due to a lack of studies regarding efficacy and safety of these drugs, there is no internationally accepted treatment approach. Therefore, current treatment of COVID-19, mainly relies on supportive attempts. However, given the burden of pandemic, obviously there is an urgent need to evaluate rational treatment options. Here it is, we tried to summarize the current conditions of anti-rheumatic drugs regarding the treatment of COVID-19 (Table 1).

### Corticosteroids

Corticosteroids (CS) are known to be strong anti-inflammatories that can suppress the host immune response, which is main responsible for developing ARDS besides, can impair the virus clearance (33). Therefore, to estimate the role of CS in the treatment of COVID-19 is challenging. Regarding the treatment of SARS, previous studies presents controversial findings, therefore WHO does not recommend routinely use of CS in the treatment of viral pneumonias (13,26).

### Non-steroid anti-inflammatory drugs (NSAIDs)

It has been demonstrated in the rats that ibuprofen induces an overexpression of ACE 2 which may lead to a viremia by presenting more binding sites to viruses (34). Therefore, to avoid to use NSAIDs may be reasonable.

### Colchicine

Basis on the colchicine prevents inflammasome assembly resulting decreased release of IL-1 and IL-6, Gandolfini et al. (32) have administered colchicine to a kidney transplant recipient with confirmed COVID-19 due to unavailability of tocilizumab, and they have noted fast improvement in the clinical and laboratory findings of the patient. An open-label, phase-2 study for colchicine is currently under review (26).

### Hydroxychloroquine

Chloroquine is able to increase endosomal pH, which is required for fusion and disrupt the virus entry via ACE 2, and it was recently shown that hydroxychloroquine (HCQ) was 3-times more potent than chloroquine (13). It was shown that chloroquine and HCQ may prevent virus entrance and inhibit viral replication in both *in vitro* and *in vivo* studies (26). In recent Chinese clinical trials, HCQ was found to be safe and effective in the treatment of COVID-19 (35). Efficacy of HCQ was demonstrated via showing a rapid viral RNA decrement in HCQ receiving patients with COVID-19 and none of them displayed long QT in their electrocardiographs (36).

### Tocilizumab

As mentioned above, SARS-CoV-2 leads to a significant inflammation resembling cytokine storm syndrome, and patients with COVID-19 were found to be with significant elevated IL-6 serum levels. Since IL-6 has a key role in this cytokine storm; tocilizumab, a blocker of IL-6, is likely to be a promising treatment option for the patients with COVID-19 (37). In a study of Xu et al, tocilizumab was found to provide significant clinical improvement without any substantial adverse effects (38). Therefore, tocilizumab has been recently added to Italian and Chinese COVID-19 management guidelines (6).

### Anakinra

Another target-specific medication candidate is anakinra, a blocker of IL-1β, which plays another central role in the pathogenesis of cytokine storm syndrome (6). Anakinra was shown to have proven efficacious in cytokine storm syndrome and macrophage activation syndrome in previous studies (39). Thus, we consider anakinra is another promising agent.

## WHAT ABOUT WITHDRAW the EXISTING or START-UP A NEW ANTI-RHEUMATIC MEDICATION?

As mentioned above, there is no sufficient evidence that patients with rheumatic disease have propensity to develop COVID-19. Moreover, it must be remembered that uncontrolled disease activity is a crucial predictor for infection among the patients with rheumatic disease (13). Therefore, rheumatologist should warn their patients for not withdrawing their medications, unless there is a contradiction (35).

Although there is a lack of data regarding start-up a new disease modifying anti-rheumatic drug or a monoclonal antibody in pandemic circumstances, in order to observe symptoms and select the available candidates, isolation of patients about 15 days and to start-up these medications to asymptomatic ones, seems like an appropriate approach (26).

In conclusion, although further studies are required, children seem to have a milder disease course and there is no sufficient evidence for the propensity of patients with rheumatic disease. Immune compromised situations of our patients and promising results of anti-rheumatic drugs present to us controversial possible outcomes. Therefore, this pandemic situation is a big challenge for we rheumatologists.

However, in the light of current *in vivo* and *in vitro* studies, we are able to say that target-specific agents, which we usually use to suppress the inflammation, are promising and there is no need to withdraw anti-rheumatic medication of our patients unless there is an additional situation.

## Figures and Tables

**Table 1:**
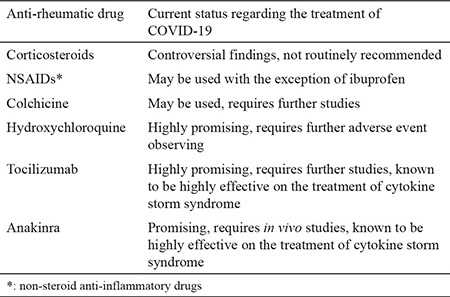
Current status of the anti-rheumatic drugs regarding the treatment of COVID-19
